# An analysis of bicycle accidents with respect to spatial heterogeneity

**DOI:** 10.1038/s41598-023-49143-9

**Published:** 2023-12-09

**Authors:** Uibeom Chun, Joonbeom Lim, Soobeom Lee, Shinhyoung Park

**Affiliations:** 1 Department of Transportation Engineering, University of Seoul, Seoul, 02504 Korea; 2 Department of Mobility Policy Research, Korea Transportation Safety Authority, Gimcheon-Si, 39660 Korea

**Keywords:** Civil engineering, Scientific data

## Abstract

Bicycles are an eco-friendly mode of transportation, and in the capital city of South Korea, Seoul, efforts are being made to encourage citizens to use bicycles. However, without appropriate safety measures, these efforts can lead to an increase in bicycle-related traffic accidents. To promote bicycle usage while ensuring safety, this study identified various factors that influence bicycle accidents. Data were utilized that had not been properly considered in previous bicycle accident-related studies, including slope and the level of public transportation services. By considering the factors influencing bicycle traffic accidents, various models were constructed, and through comparisons of statistical indicators, the optimal model was selected geographically weighted negative binomial regression. Ultimately, three significant conclusions to ensure bicycle safety were drawn. First, across all areas of Seoul, an increase in road slope leads to a decrease in bicycle-related accidents. Furthermore, for certain Traffic Analysis Zones (TAZs), as the number of local buses (or neighborhood/community buses) increases, the bicycle traffic volume decreases, resulting in a reduction in bicycle accidents. Lastly, for some TAZs, an increase in bicycle lanes to be installed into the roadway was associated with an increase in bicycle accidents.

## Introduction

Bicycles are sustainable and eco-friendly modes of transportation. They contribute to alleviating urban congestion and environmental pollution resulting from urbanization trends, and offer potential improvements to public health^1,2^. Bicycles have gained traction globally as a mode of transport, with active policies implemented across Europe and in various cities around the world, including Korea, where sharing bike programs and expanded bicycle lanes are common. However, despite these efforts, a survey conducted by the digital insurance company Luko in 2023 revealed that among 90 cities worldwide, Seoul ranked 71st with a bicycle usage rate of 1.5%. This low ranking places Seoul behind other cities of similar size, such as Tokyo (27%), Madrid (6%), Cairo (5%), and Santiago (3.9%). Though there are multiple reasons for the lack of preference for bicycles in Seoul, a survey by Hankook Research^3^ found that bicycle users feel more threatened on the roads compared to typical vehicle users. Over the past 5 years (2017–2022), the total number of accidents in Seoul has decreased by an average of 2.4% annually, and the number of injured individuals has decreased by an average of 3.4% annually. In contrast, over the same period, the number of accidents related to bicycles has increased by an average of 3.1% annually, and the number of injured individuals has increased by an average of 3.8% annually. To promote bicycle usage, ensuring safety for cyclists is paramount. Thus, understanding the relation between factors that significantly influence bicycle accidents is crucial.

Based on previous research, this study categorized the influential factors behind bicycle accidents into Demographic & Socio-economic Variables, Road Operation & Management Variables, Road-Infrastructure Variables, and Spatial Interaction.

Regarding Demographic & Socio-economic Variables, it was found that bicycle accidents increased with an increase in population or population density^4–8^. Age group proportions also significantly influenced bicycle-accident numbers. Younger age groups (under 19 years old), who often use bicycles for limited travel purposes, showed a decreased accident rate due to their lower usage^7^. Conversely, for age groups over 18 years old, a higher proportion of the population was associated with an increase in bicycle accidents^8^. Furthermore, males had a higher likelihood of being involved in accidents due to traffic violations compared to females^9^.

In the analysis of Road Operation & Management Variables, among variables such as traffic volume and road length, which have the greatest impact on general vehicle accidents, bicycle traffic accidents were positively correlated with traffic volume^10–12^, Bicycle Distance Traveled (BDT), and Bicycle Time Traveled (BTT)^2,13,14^. Bicycle lanes, installed to ensure the passage of bicycle users, showed a negative correlation with bicycle accidents^15^. This suggests that even with an increase in users after the installation of bicycle lanes, accidents did not increase significantly due to their presence^15^. However, some research have shown that in certain road environments, such as arterial roads with high traffic volume, an increase in bicycle lane length leads to an increase in accidents^16^. Additionally, there was a statistically significant increase in bicycle accidents with an increase in signalized intersections^5^. As bicycles often play a role in providing the “First-mile” and “Last-mile” connections for public transportation users, subway stations with high demand were found to increase bicycle accidents, and an increase in bus stops near subway stations also showed a corresponding increase in bicycle accidents^17^. Moreover, bicycle users were more likely to meet with accidents in areas with high bus stop and bus route densities^18^. The increased density of bus stops could result in interactions between different road users, thus potentially raising the likelihood of accidents for bicycle users^19^.

Under the Road-Infrastructure variables, road width and road slope are significant factors. Studies examining the influence of road width on bicycle accidents show conflicting results. Some suggest that as the proportion of roads with widths exceeding 55 m increases, accidents decrease^18^. Other studies indicate that designing roads with narrower widths could potentially reduce bicycle accidents^20^. As for road slope, an increase in slope is associated with a decrease in bicycle traffic accidents^16^. This could be attributed to the negative impact of steeper slopes on cyclists’ route choices^21^, leading to a decrease in bicycle usage on roads with high slopes, which subsequently results in fewer accidents.

In previous studies, various factors resulting in bicycle accidents have been identified; however, there are limitations in the results’ interpretation. The first limitation is that the impact of the independent variables on the number of bicycle accidents has been estimated in the form of fixed parameter (coefficient values). However, the influence of the same variables on the number of accidents can vary depending on the characteristics of the area^22,23^. Traditional traffic accident frequency or severity prediction models used in previous research, such as the negative binomial model^4,12,15^, logit model^9^, and multivariable conditional logistic regression^11^, have fixed parameters, which means they cannot account for changes over time and spatial heterogeneity.

To overcome these limitations, models such as the random parameter model^7,24^ and random effect model^16,18^ have been used. These models have flexible parameters and offer the advantage of interpreting results based on spatial characteristics. However, these models have a limitation in that while the coefficients of variables exist within a range, they cannot specify which coefficient values belong to a particular space. To address this limitation, spatial analysis methods have been employed. The concept of spatial analysis is explained by the first law of geography: “Everything is related to everything else, but near things are more related than distant things”. Given that traffic accidents are spatial phenomena, many studies argue for the existence of spatial interaction among accidents^5,16,25,26^. This spatial analysis has evolved by combining traditional frameworks such as the Poisson model and Negative Binomial model with spatial elements, leading to models like GWPR^27,28^ and GWNBR^26^. These models allow for the interpretation of spatial heterogeneity for each variable and enable the identification of variations in the significance of variables across particular spatial areas.

In this study, data was collected on factors influencing bicycle accidents, as presented in previous research, for 424 TAZ (Traffic Analysis Zone) located within Seoul. Additionally, variables were identified that had not been thoroughly considered before, such as road-centric slope and the level of public transportation service. The analysis involved a comparison of goodness-of-fit and impact variables for bicycle accidents across different models. These models encompassed the Negative Binomial model (NB), which effectively accounts for over-dispersed data, the Random Parameter Negative Binomial model (RPNB), designed to address unobserved heterogeneity and over-dispersion, and the Geographic Weighted Negative Binomial Regression(GWNBR) that be able to accommodate spatial heterogeneity and over-dispersed data.

## Materials and methods

### Estimating the slope of roadways

In existing literature, two methods have primarily been used to calculate the representative slope within an area unit like TAZ. The first method involves calculating the zonal average slope, which calculates the average slope of the terrain within the analyzed area^16^. The second method, called the average weighted slope, calculates the average slope taking into account the slope and length of each road link within the area^1,29^. To derive this, the formula shown in Eq. (1) is applied:

1 $$Average\;Weighted\;Slope\;in\;TAZ= \frac{({l}_{1}*{s}_{1}+{l}_{2}*{s}_{2}+\dots +{l}_{n}*{s}_{n})}{\sum_{i}^{n}{l}_{i}}$$

In the equation, ${l}_{i}$ represents the length of each segment and ${s}_{i}$ represents the slope of each segment. Using the mentioned variables, it is possible to determine the average weighted slope of a road within a specific TAZ or segments. The average weighted slope method has the advantage of considering not only the slope of each road link but also its length when compared to the zonal average slope method. However, applying these methods to this study presents two issues.

**Figure 1:**
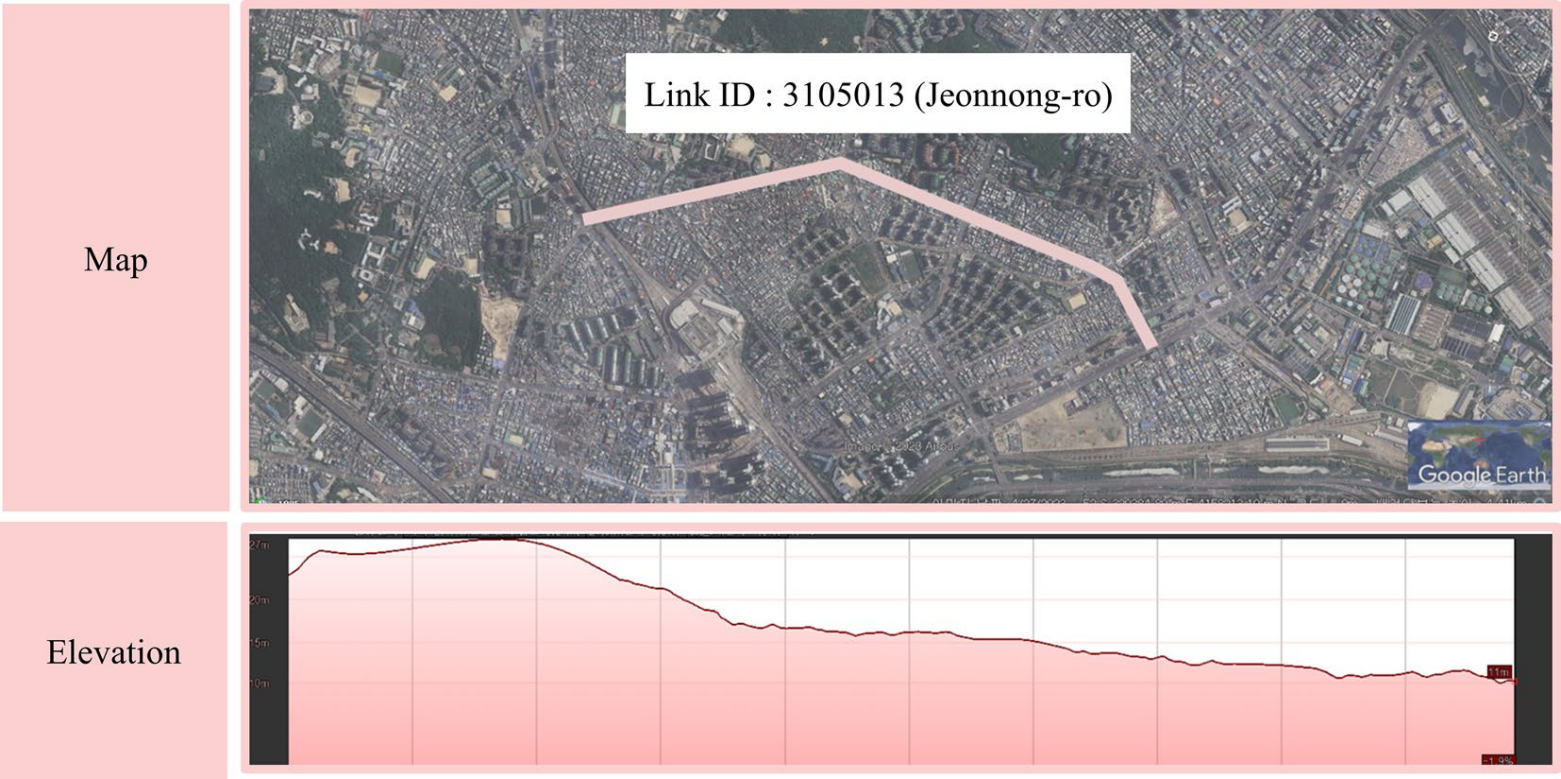
Slope of road in Seoul.

First, these methods cannot account for the unique topographical characteristics of Seoul, the spatial scope of this study. Seoul exhibits significant variations in elevation, ranging from 0 to 752 m. Thus, calculating the zonal average slope, which considers the overall terrain slope within the simple analysis unit (such as TAZ), might yield incorrect values by estimating the slope of terrain where roads are not actually present. Moreover, the average weighted slope method, which calculates slope on a per-link basis, is unsuitable for this study due to frequent elevation changes within individual road links (Fig. 1). This may offset the overall slope of the road where the change in slope is large.

Second, during the process of deriving the representative slope within the analysis unit, both methods could produce inaccurate results. The zonal average slope might overestimate the representative slope due to its use of absolute values, which prevents distinguishing between uphill and downhill slopes. Moreover, the average weighted slope method might converge the representative slope to zero if uphill and downhill slopes within a link have the same length and slope.

Though the average weighted slope method has advantages in considering link length and non-terrain slopes. These two methods produce difficulties when applied to the unique topography of Seoul and might lead to inaccurate results during the aggregation process to derive representative slopes.

**Figure 2:**
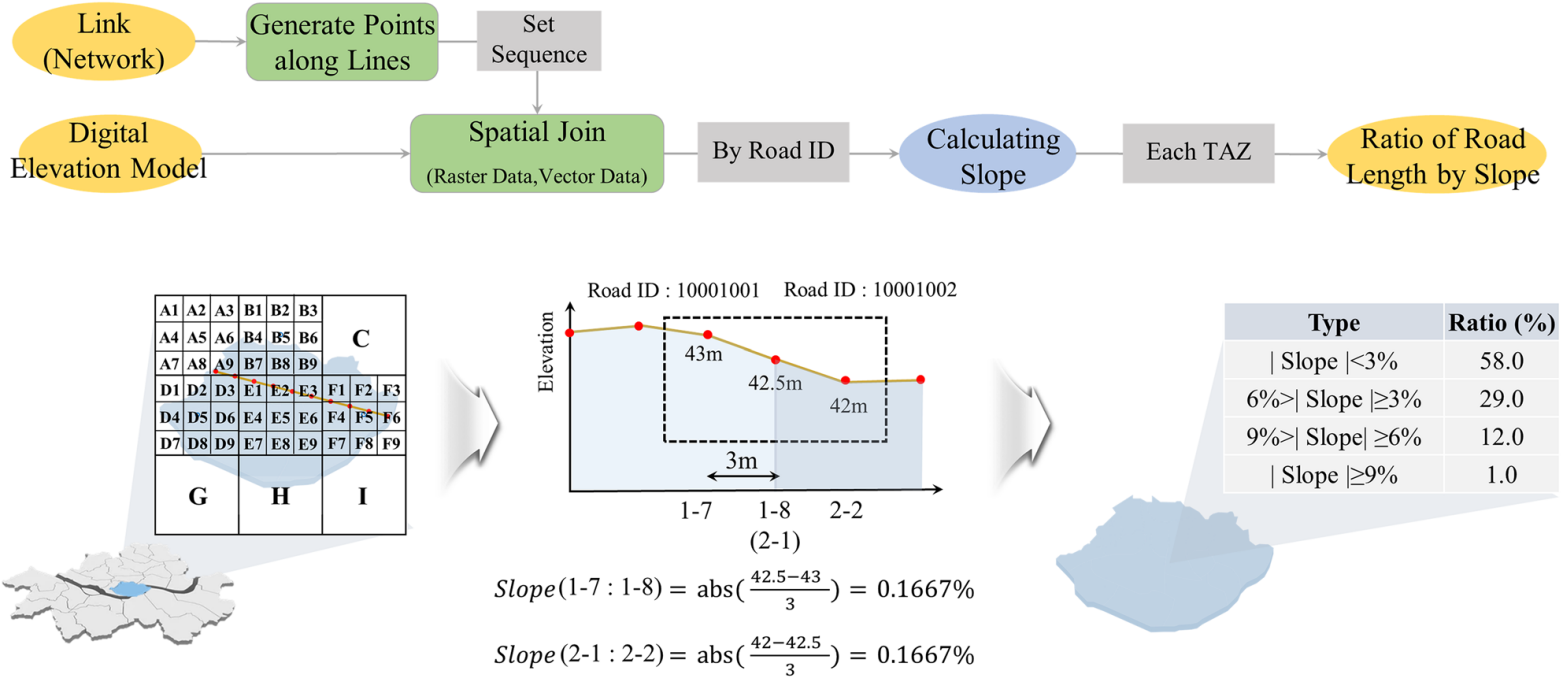
Flow of estimating of slope in this study.

To overcome these limitations, the following methods have been followed in this study (Fig. 2). First, considering the specifications of bicycles, points at 3-m intervals were generated for each link. Thereafter, spatial joining was performed with a Digital Elevation Model (DEM) that includes elevation information. Through this process, elevation values were assigned to each point. Next, the slope between adjacent points was calculated based on the elevation difference and distance (3 m), thus establishing a variable representing the percentage of road length with respect to gradient categories within the analysis unit. This method enables the calculation of gradients at the level of individual link segments and the overcoming of data smoothing issues that may occur during the aggregation process.

### Level of public transportation service supply

The analysis of the relationship between service supply and bicycle accidents in previous studies primarily relied on metrics such as the number of stations and the density of bus stops^17–19^. However, considering only the infrastructure of public transportation could lead to erroneous results. For instance, even if a specific area has a high density of bus stops, differences in bus frequencies and intervals between stops could result in a low level of service. Additionally, the presence of subway stations within an area does not necessarily reflect good service if the distance between residential areas and the stations is substantial^30^. Relying solely on the number of subway stations could lead to biased conclusions.

To avoid such biases, this study quantifies the level of actual public transportation service provided to citizens. This is achieved by dividing the service level into spatial and temporal aspects. The spatial service level is calculated by assessing the ratio of road length accessible by public transportation infrastructure (bus stops, subway stations) within each TAZ compared to the total road length. A higher ratio indicates a higher spatial service level of public transportation in that TAZ. Roads accessible by public transportation refer to roads that can be reached on foot from each facility, with walking distances varying based on land use categories (residential, commercial, industrial, green) in accordance with the National Land Planning and Utilization Act, Korea. Walking distances for residential, commercial, and industrial zones were set at 400 m from bus stops and 800 m from subway stations, whereas for green zones, both bus stops and subway stations were set at 800 m. The distances were calculated using the Manhattan distance method, which considers the actual road layout rather than the Euclidean method.

The temporal service level considers the ratio of stations satisfying a minimum service frequency criterion compared to the total number of stations within the target area. The minimum service frequency criterion was established based on population density quantiles (25th percentile: 200 people/km^2^, 75th percentile: 8350 people/km^2^) from the 2019 TAZ data. This criterion was thereafter applied to each station using the guidelines presented^31^. Specific criteria for calculating the transit service level are outlined in Table 1.

**Table 1:** Transit service (spatial, temporal) satisfaction criteria.

Spatial service	
Characteristic of landuse	Range of adopted public transportation service
Residential area, commercial area, industrial area	Bus stop: 400 m
	Subway station: 800 m
Green area	Bus stop: 800 m
	Subway station: 800 m

Temporal service	
Density of population	Number of trips by stop
Low: less than 200 people/km^2^	1 trip per 1 h
Medium: 200–,8350 people/km^2^	1.33 trips per 1 h
High: over 8350 people/km^2^	6 trips per 1 h

Source: Korea Transportation Safety Authority, Transit Capacity and Quality of Service Manual.

**Figure 3:**
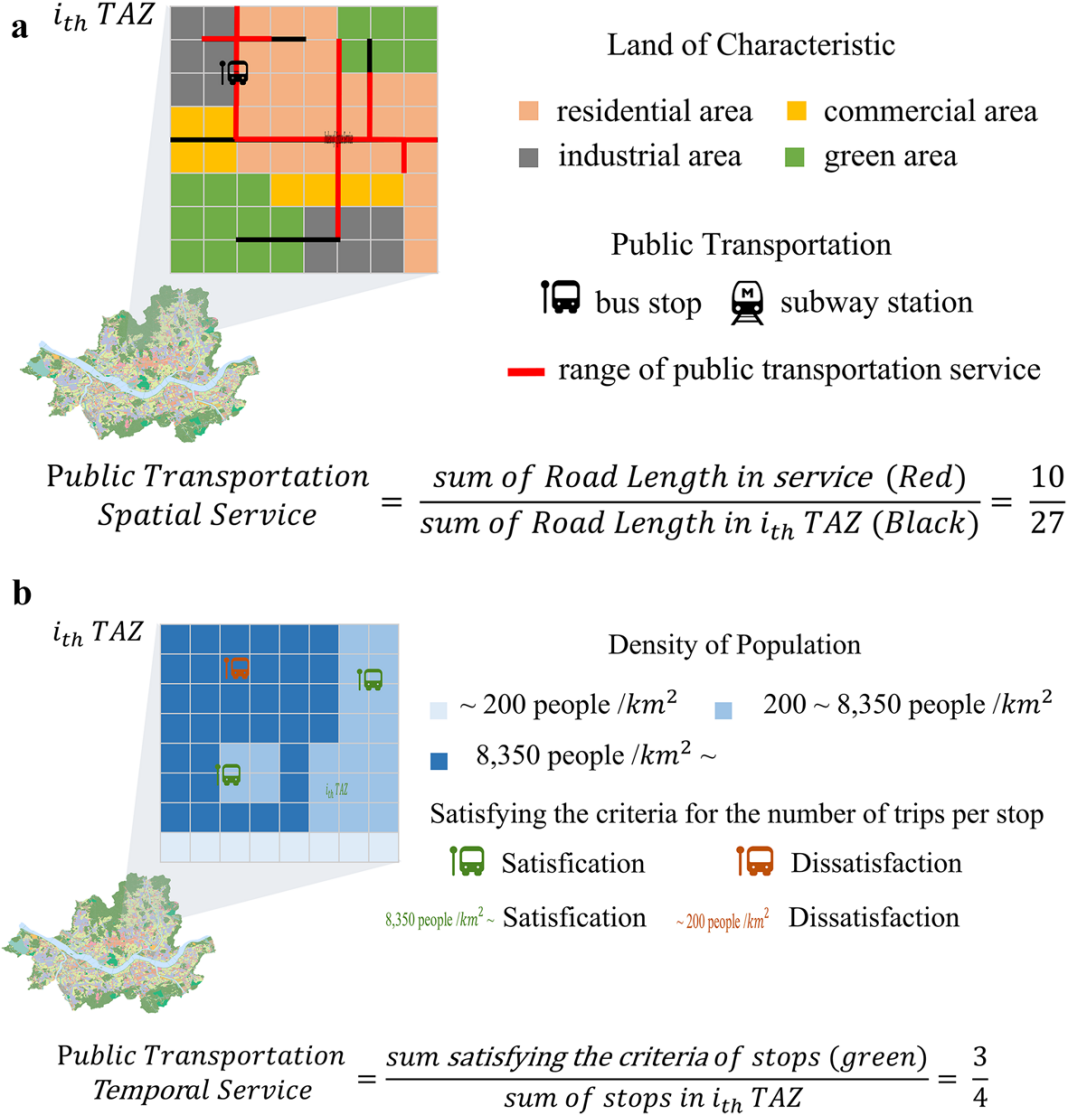
(**a**) Method of estimating public transportation spatial service. (**b**) Method of estimating public transportation temporal service.

Figure 3-a,b present the method used for ultimately calculating public transportation service level of spatial and temporal services, according to the criteria presented in Table 1.

### Estimation of traffic accidents according to global and local models

Models are categorized into global models and local models based on the assumption of spatial stationarity to quantitatively assess and predict the occurrence of traffic accidents^27,32^. Spatial stationarity refers to the assumption that the impact of independent variables on the dependent variable remains consistent regardless of location. Global models assume this spatial stationarity and aim to explain the entire analysis domain with a single regression equation^27^. Previous studies that used global models to explain traffic accidents considered the characteristics of accident data as count data and the over-dispersion of accident counts collected at different analysis units compared to their mean. Thus, they employed techniques such as Poisson regression and negative binomial regression^14,27,28^. Additionally, some studies tackled the issue of excessive zero counts due to the majority of the analysis units having no accidents by using zero-inflated regression^33^.

However, local models assume spatial heterogeneity, which means that the statistical properties of data vary due to inherent characteristics of specific points or regions in space. As traffic accidents occurring on roads exhibit spatial heterogeneity, failing to consider this in constructing accident prediction models can lead to biased results due to data skewness^26,34^. A prominent model for considering spatial heterogeneity is the GWR model, which accounts for spatial heterogeneity by generating distinct local regressions for each observation point. The GWR model, being based on linear model (OLS), has limitations when dealing with count data that takes non-negative values or data with over-dispersion^35,36^. As a result, some studies have employed GWNBR to simultaneously consider spatial heterogeneity and over-dispersion in the dependent variable^26,28^. They argued that GWNBR outperforms models that do not account for over-dispersion, particularly in cases where data exhibit over-dispersion, offering improved predictive power and accuracy. GWNBR extends the global Negative Binomial regression by allowing for parameter and spatial variations. The basic equation of GWNBR is presented below (2).

2 $$Y_{i} \left( {v_{i} ,u_{i} } \right) = NB\left[ {t_{i} {\text{exp}}\left( {\sum\limits_{{k = 1}}^{n} {\beta _{k} } \left( {v_{i} ,u_{i} } \right)X_{{ik}} } \right),\alpha \left( {v_{i} ,u_{i} } \right)} \right]$$

${Y}_{i}\left({v}_{i},{u}_{i}\right)$ denotes the dependent variable at $\left({v}_{i},{u}_{i}\right)$; ${X}_{ik}$ denotes the Kth independent variable at $\left({v}_{i},{u}_{i}\right)$; ${\beta }_{k}\left({v}_{i},{u}_{i}\right)$ denotes the regression coefficient of ${X}_{ik}$; $\alpha \left({v}_{i},{u}_{i}\right)$ denotes over-dispersion at $\left({v}_{i},{u}_{i}\right);$ and ${t}_{i}$ denotes the offset variable.

### Data

To identify significant factors influencing bicycle accidents, data were collected over a three-year period (2017–2019) for bicycle traffic accidents that occurred throughout Seoul’s 424 TAZ. The collected the accident data were gathered using the TAAS (Traffic Accident Analysis System) service provided by the Korea Road Traffic Authority (https://taas.koroad.or.kr/). Accidents data and road data were each organized into shapefiles (shp), and spatial analysis was performed using the GIS software. The resulting preprocessed dataset was thereafter collected in the research. Next, factors found in previous studies to significantly influence bicycle accidents were categorized into five groups and collected for each TAZ: (1) Demography related variables, (2) Traffic volume related variables, (3) Road characteristic related variables, (4) Road enforcement related variables, and (5) Accessibility related variables.

Demography related variables were obtained from Statistics Korea (https://kostat.go.kr/) and the official website of Seoul (https://data.seoul.go.kr/). The age groups were divided into 15–64 years to match the available range of data from 2017 to 2019, aligning with the age of usage for the public bicycle service “Ttareungyi,” which requires riders to be 15 years and older, the common retirement age in Korea being 65 years.

Traffic volume related variables were acquired from KTDB (Korea Transport Database, https://www.ktdb.go.kr/), which provides data on the volume of traffic entering and exiting each TAZ on an annual basis for various transportation modes.

Road characteristic related variables, including small-scale roads like community roads, were obtained from the Road Name Address site (https://www.juso.go.kr/) and combined with Digital Elevation Model (DEM) data from JPL (NASA-Jet Propulsion Laboratory, https://asterweb.jpl.nasa.gov/) to calculate road slopes.

Road enforcement related variables and Accessibility related variables were collected using sources such as the official website of the Seoul (https://data.seoul.go.kr/) and the Korea Transportation Safety Authority (https://www.kotsa.or.kr/). In total, 23 variables were constructed, as shown in Table 2.

**Table 2:** Description of variables used in this study.

Variables	Description	Min	Max	Mean	SD
Crash related variable [a]					
Number of crash	Bicycle crash count in each TAZ	0	86	17.20	13.77
Demographic related variable [b, c]					
Number of people	Log of the number of all people in each TAZ	2.93	4.75	4.32	0.22
Male	The percentage of male in each TAZ	42.23	61.93	49.02	1.92
Female	The percentage of female in each TAZ	38.07	57.77	50.97	1.92
People_15	The percentage of people (under 15 years old) population in each TAZ	3.13	24.21	10.49	3.18
People_15–64	The percentage of people (15–64 years old) population in each TAZ	66.13	88.30	74.56	2.92
People_65+	The percentage of people (over 65 years old) population in each TAZ	6.44	26.51	14.95	3.08
Traffic volume related variable [d]					
Total traffic volume	Log of the annual average daily total traffic in each TAZ	9.11	13.10	10.90	0.69
Local bus volume	Log of the annual average daily traffic of local bus in each TAZ	3.00	9.27	6.60	1.02
Bicycle volume	Log of the annual average daily traffic of bicycle in each TAZ	0.00	9.97	7.24	1.75
Road characteristic related variable [e, f]					
Road length	Log of the road length in each TAZ (m)	7.75	11.00	9.75	0.56
Road_0	The percentage of road (slope < 3%) in each TAZ	20.65	99.80	73.21	19.79
Road_3	The percentage of road (slope > 3%, slope < 6%) in each TAZ	0.12	53.25	19.91	13.56
Road_6	The percentage of road (slope > 6%, slope < 9%) in each TAZ	0.00	15.26	2.37	2.89
Road_9+	The percentage of road (slope > 9%) in each TAZ	0.00	36.37	4.46	5.53
Bicycle path	The percentage of bicycle path in each TAZ	0.00	82.75	9.80	12.91
Traffic light	The number of traffic light by road per 1km in each TAZ	0.00	34.88	17.50	3.53
Traffic warning light	The number of traffic warning light by road per 1km in each TAZ	0.00	6.29	3.10	0.44
Traffic pedestrian light	The number of traffic pedestrian light by road per 1km in each TAZ	0.00	4.51	2.37	0.43
Road enforcement related variable [c]					
Illegal parking	The number of illegal parking crackdown per 1km in each TAZ	0.22	1745.09	70.62	151.50
Complaint	The number of complaints per 1km in each TAZ	0.55	486.20	55.30	69.61
Accessibility related variable [g]					
Spatial service	Level of public transportation spatial service in each TAZ	0.62	1.00	0.97	0.06
Temporal service	Level of public transportation temporal service in each TAZ	0.21	1.00	0.85	0.17

Reference of [a]: Korea Road Traffic Authority (https://taas.koroad.or.kr/).

Reference of [b]: Statistics Korea (https://kostat.go.kr/).

Reference of [c]: Official website of the Seoul (https://data.seoul.go.kr/).

Reference of [d]: KTDB (Korea Transport Database, https://www.ktdb.go.kr/).

Reference of [e]: Ministry of the Interioir and Safety (https://www.juso.go.kr/).

Reference of [f]: JPL (https://asterweb.jpl.nasa.gov/).

Reference of [g]: Korea Transportation Safety Authority (https://www.kotsa.or.kr/).

**Figure 4:**
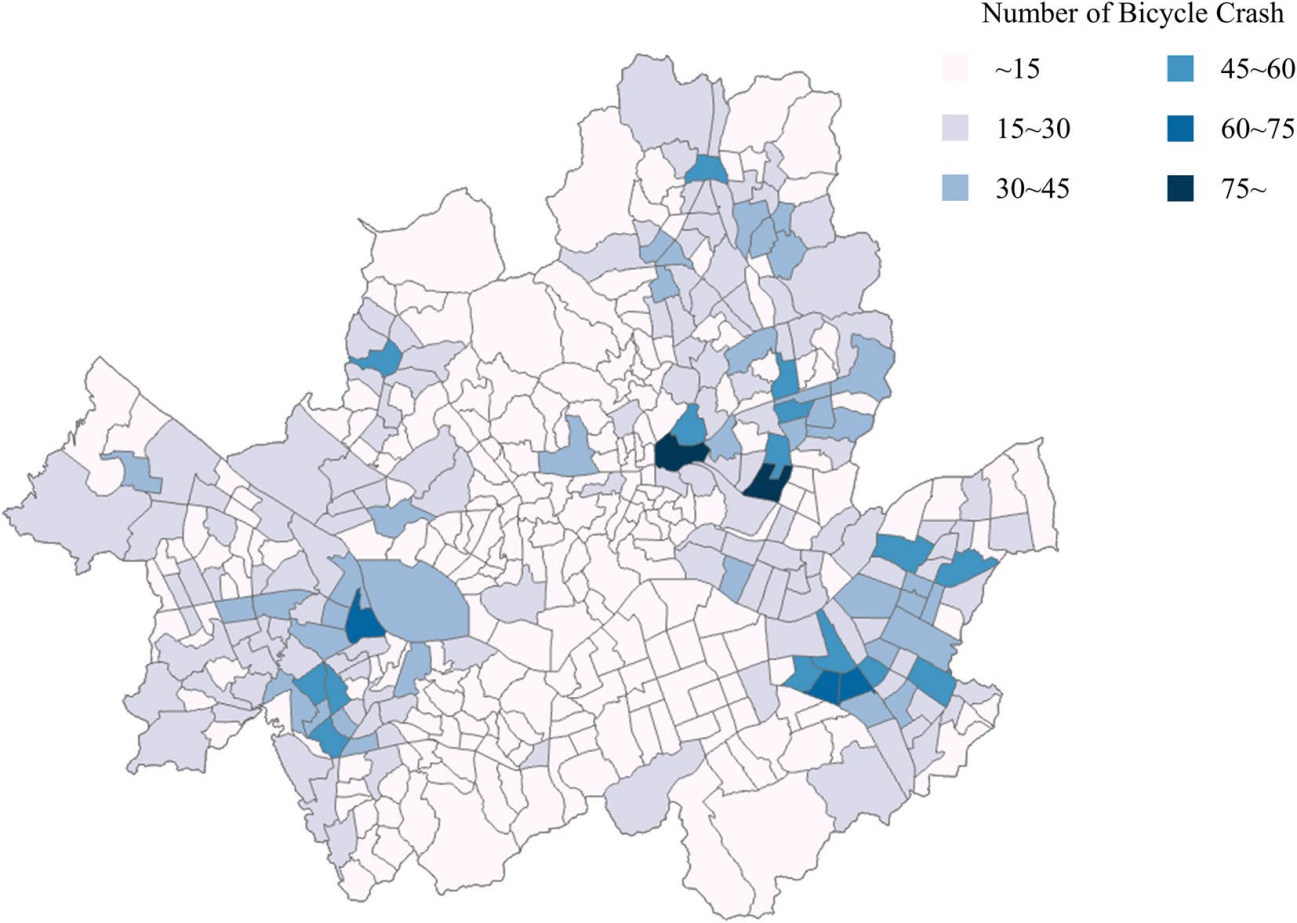
The total bicycle crash in Seoul in 3 years (2017–2019).

Figure 4 illustrates the visualization of bicycle accidents that occurred during the study period, using Geographic Information System (GIS) tools and shows that all 424 TAZs exhibit varying bicycle accident counts, indicating significant differences among TAZs in terms of accident frequency. In Fig. 4, when examining the distribution of bicycle traffic accidents, it is evident that accidents are concentrated around the southwestern and eastern regions.

All events occurring in space exhibit interactions with each other, influencing the spatial distributions among neighboring areas^37^. This spatial interaction is classified characteristics of both spatial dependence and spatial heterogeneity. Spatial dependency implies that events at a specific location "A" have more similar characteristic to nearby occurrences than those situated farther away^38,39^. Spatial heterogeneity implies that the impact of the same variable on an event can vary due to geographical differences in location^38,39^. In adherence to the concept of spatial dependence, events at a given location "A" share similarities with neighboring events, whereas events occurring at a considerable distance from "A" exhibit somewhat less resemblance to those at A. This phenomenon gives rise to spatial heterogeneity. Spatial interaction, also termed spatial autocorrelation, has been commonly assessed through the Moran’s I test in previous studies^5,7,25,32^. The Moran’s I test determines whether specific events exhibit spatial clustering or dispersion. The resulting Moran’s I index, ranging from − 1 to + 1, indicates the degree of clustering, with a value closer to + 1 suggesting a more clustered distribution. In the context of this study, the application of Moran’s I has a statistically significant Moran’s I index of 0.42 (p-value: 0.000, means significant at confidence level 99%) like Table 3. This result indicates a pronounced spatial interaction among the traffic accident data analyzed, therefore, suggests to account for spatial heterogeneity, given the nature of traffic accidents occurring in space.

**Table 3:** Moran’s I statistics for analysis data.

Type	Index	Z-score	P-value
Moran’s I	0.42	14.61	0.00

## Results

This study developed four models for explaining bicycle accidents that occurred in Seoul’s each administrative district, based on the distribution of the data collected. Their fit and predictive powers were compared, and the optimal model that performed best was selected, after which the results were interpreted.

### Correlation between variables

Pearson’s correlation coefficients were used to analyze correlations between the data collected, the results of which are presented in Table 4. This confirmed a high correlation between certain explanatory variables. The male to female ratio showed a perfectly negative correlation (− 1.00); thus, only the ratio of men was considered in later analyses. For the ratio of roads classified by slope, there were strong negative correlations (− 0.95, − 0.88, − 0.79) between cases wherein the slope size was 3% or lower, and more than 3%. Moreover, there was a strong positive correlation between road ratio variables of slope size exceeding 3%. Thus, this study only maintained ROAD_3 among the four variables related to road slope, and excluded all others. Next, a strong positive correlation (0.99) was also observed between the number of traffic lights and pedestrian signals per kilometer in each TAZ; thus, variables related to pedestrian signals were excluded in later analyses. Therefore, certain variables were eliminated for solving the problem of multicollinearity, which may occur due to high correlations between some independent variables.

**Table 4:** Correlation coefficients matrix.

Variables	NC	NP	M	F	P15	P1564	P65	TTV	LBV	BV	RL	R0	R3	R6	R9	BR	TL	TWL	TPL	IP	C	TS	SS
Number of crash (NC)	1.00																						
Number of people (NP)	**0.37**	1.00																					
Male (M)	**0.12**	**− 0.23**	1.00																				
Female (F)	**− 0.12**	**0.23**	**− 1.00**	1.00																			
People_15 (P15)	**− **0.04	**0.36**	**− 0.33**	**0.33**	1.00																		
People_15–64 (P1564)	**0.16**	**0.04**	**0.12**	**− 0.12**	**− 0.49**	1.00																	
People_65+ (P65)	**− 0.11**	**− 0.42**	**0.23**	**− 0.23**	**− 0.56**	**− 0.44**	1.00																
Total traffic volume (TTV)	**0.32**	**0.24**	0.05	**− **0.05	**− **0.03	**0.23**	**− 0.19**	1.00															
Local bus volume (LBV)	**− 0.06**	**0.12**	**− **0.03	0.03	**− **0.07	**0.08**	0.00	**0.42**	1.00														
Bicycle volume (BV)	**0.46**	**0.30**	**0.15**	**− 0.15**	0.00	**0.22**	**− 0.21**	**0.45**	**0.14**	1.00													
Road length (RL)	**0.01**	0.05	**− **0.03	0.03	0.02	**− **0.02	**− **0.01	**− **0.01	**− **0.02	0.03	1.00												
Road_0 (R0)	**0.57**	**0.12**	**0.08**	**− 0.09**	**0.08**	**0.13**	**− 0.20**	**0.20**	**− 0.12**	**0.49**	0.02	1.00											
Road_3 (R3)	**− 0.56**	**− 0.09**	**− **0.07	0.07	**− 0.08**	**− 0.08**	**0.16**	**− 0.17**	**0.12**	**− 0.44**	**− **0.02	**− 0.95**	1.00										
Road_6 (R6)	**− 0.47**	**− 0.13**	**− 0.11**	**0.11**	**− **0.03	**− 0.18**	**0.19**	**− 0.21**	**0.11**	**− 0.48**	**− **0.02	**− 0.88**	**0.73**	1.00									
Road_9+ (R9)	**− 0.42**	**− 0.15**	**− **0.07	0.07	**− **0.06	**− 0.18**	**0.23**	**− 0.21**	**0.09**	**− 0.43**	0.00	**− 0.79**	**0.56**	**0.84**	1.00								
Bicycle path (BR)	**0.20**	0.06	**− 0.14**	**0.14**	**0.39**	**− 0.10**	**− 0.30**	**0.18**	**− **0.06	**0.27**	0.01	**0.31**	**− 0.32**	**− 0.23**	**− 0.21**	1.00							
Traffic light (TL)	**0.36**	**0.35**	0.01	**− **0.01	**0.22**	**− **0.06	**− 0.17**	**0.54**	**0.22**	**0.35**	**0.10**	**0.23**	**− 0.23**	**− 0.20**	**− 0.16**	**0.36**	1.00						
Traffic warning light (TWL)	**0.12**	**0.10**	**− **0.01	0.00	0.01	0.02	**− **0.02	0.03	0.03	0.06	0.01	0.00	0.00	0.00	0.01	0.02	0.06	1.00					
Traffic pedestrian light (TPL)	**0.38**	**0.34**	0.02	**− **0.02	**0.21**	**− **0.04	**− 0.18**	**0.52**	**0.20**	**0.37**	**0.09**	**0.27**	**− 0.27**	**− 0.24**	**− 0.19**	**0.38**	**0.99**	0.01	1.00				
Illegal parking (IP)	**0.09**	**− **0.03	**− **0.01	0.01	0.00	0.01	**− **0.01	**0.28**	**0.10**	0.01	**− 0.30**	**0.08**	**− **0.07	**− **0.06	**− 0.08**	0.06	**0.22**	0.01	**0.20**	1.00			
Complaint (C)	**0.08**	0.02	**− **0.03	0.03	**− 0.15**	**0.23**	**− **0.06	**0.32**	**0.17**	0.06	**− 0.47**	0.04	**− **0.03	**− **0.03	**− **0.05	**− **0.02	**0.17**	0.02	**0.16**	**0.55**	1.00		
Spatial service (SS)	**− **0.02	0.04	0.06	**− **0.06	**− **0.04	0.06	**− **0.02	0.05	**0.19**	**0.08**	0.01	0.00	0.04	**− **0.04	**− 0.08**	**− **0.06	0.03	0.02	0.04	0.02	0.01	1.00	
Temporal service (TS)	**− **0.06	**− 0.15**	0.02	**− **0.02	**− 0.08**	0.02	0.06	0.06	**− 0.08**	0.03	0.06	0.00	**− **0.02	0.02	0.03	0.07	0.04	**− **0.01	0.03	**− **0.01	**− **0.01	**− 0.10**	1.00

Bold: Correlation is significant at the 0.1 level.

### Selection optimal model

In order to identify factors influencing bicycle accidents within the analysis scope, it is necessary to utilize the optimal model that best predicts the occurrence of bicycle traffic accidents. Due to the over-dispersion in the variance (189.61) of the constructed bicycle accident counts by TAZ, which is greater than the mean (17.20), models based on the negative binomial distribution were constructed. Due to geographical and characteristic variations in the dataset, reflecting spatial heterogeneity was essential. Thus, model capable of addressing over-dispersion and heterogeneity, namely the Random Parameter Negative Binomial (RPNB) as well as model capable of addressing over-dispersion and spatial heterogeneity, GWNBR, were constructed. The results of constructing the RPNB model showed that only two variables with heterogeneity (road slope of 9% or more, temporal accessibility of public transit services) were significant. The mean coefficient value for the variable of road slope 9% or more was − 8.313, indicating a decrease in bicycle accidents, and the scale parameters were 3.103, showing a range of − 14.519 to − 2.107 (mean parameter ±  2 scale parameter (δ)). Additionally, temporal accessibility of public transportation services showed a mean coefficient value of − 0.528, indicating a decrease in bicycle accidents, and the scale parameters were 0.218, showing a range of − 0.964 to − 0.092 (mean parameter ±  2 scale parameter (δ)). Although the RPNB model can probabilistically calculate the coefficient values of variables affecting bicycle traffic accidents and produce a range, it has the limitation of being unable to specify the coefficient values in a particular space. To select the model with the highest predictive power and accuracy among them, evaluation metrics were utilized, including AICc, *R*^2^, Adjusted *R*^2^, RMSE (Root Mean Squared Error), and MAPE (Mean Absolute Percentage Error). Tables 5, 6 and 7 present the results each constructed model in this study.

**Table 5:** Summary results of the global model for bicycle crash (NB).

Negative binomial model (NB)				
Variables	Coefficient	Std. Error	Z-value	P-value
Number of people	1.306	0.139	9.370	0.000
People_15–64	4.524	0.960	4.712	0.000
People_65+	6.723	0.970	6.937	0.000
Total traffic volume	0.157	0.052	2.986	0.002
Local bus volume	− 0.070	0.015	− 4.600	0.000
Bicycle volume	0.139	0.031	4.458	0.000
Road_3	− 2.429	0.238	− 10.190	0.000
Road_9+	− 3.008	0.674	− 4.462	0.000
Traffic light	0.021	0.006	3.194	0.001
Temporal service	− 0.357	0.143	− 2.502	0.012
Model fit statistic				
AICc	2817.800			
${R}^{2},$ ${Adj R}^{2}$	0.612, 0.602			
MAPE	0.402			
RMSE	8.581

**Table 6:** Summary results of the random parameter negative binomial model for bicycle crash (RPNB).

Random parameter negative binomial model (RPNB)				
Variables	Coefficient	Std. error	t-ratio	P-value
Non-random parameters				
Number of people	2.437	0.514	4.745	0.000
People_15–64	− 0.786	0.417	− 1.883	0.059
People_65+	0.523	0.069	7.554	0.000
Local bus volume	− 0.066	0.008	− 8.366	0.000
Bicycle volume	0.250	0.019	13.407	0.000
Traffic light	0.038	0.004	9.784	0.000
Means for random parameters				
Road_9+	− 8.313	0.381	− 21.844	0.000
Temporal service	− 0.528	0.101	− 5.214	0.000
Scale parameters for dists. of random parameters				
Road_9+	3.103	0.306	10.157	0.000
Temporal service	0.218	0.020	10.839	0.000
Dispersion parameter for negative binomial distribution				
ScalParm	5.265	0.317	16.600	0.000
Model fit statistic				
AICc	2925.29

**Table 7:** Summary results of the local model for bicycle crash (GWNBR).

Geographic weighted negative binomial regression (GWNBR)				
Variables	Coefficient			
	Min	Max	Mean	SD
Number of people*	0.760	1.744	1.395	0.209
People_15–64*	0.517	9.836	5.902	2.602
People_65+*	− 0.326	11.486	8.047	2.493
Total traffic volume*	0.027	0.349	0.144	0.072
Local bus volume*	− 0.162	0.039	− 0.039	0.046
Bicycle volume*	0.007	0.256	0.144	0.059
Road_3*	− 3.448	− 1.750	− 2.948	0.329
Bicycle path*	− 0.483	1.298	0.419	0.519
Traffic light*	− 0.011	0.046	0.022	0.014
Temporal service (90% confidence)	− 0.747	− 0.132	− 0.358	0.118
Bandwidth	5.111			
Model fit statistic				
AICc	2778..467			
${R}^{2},$ ${Adj R}^{2},$	0.714, 0.707			
MAPE	0.350			
RMSE	7.376			

*Significant at a 95% confidence level.

Each tables include explanatory variables and their coefficients that were found to be statistically significant within a 95% confidence level for predicting bicycle accident counts using each respective model. Additionally, the tables present statistical indicators representing the models’ predictive power and accuracy. The significance of individual variables was determined using the t-value. After comparing the evaluation metrics obtained from the different models, the GWNBR model, which considers both over-dispersion and spatial heterogeneity, exhibited the best results across all evaluations. Thus, GWNBR model was selected for this study.

## Discussion

It is observed that the significant variables in NB model align with the significant variables in GWNBR model. However, in the GWNBR model, the coefficient values of variables provide a range from minimum to maximum, which can be include coefficient values opposite in sign to those in NB model. In other words, variables that increase bicycle accidents in NB model may, in GWNBR model, decrease accidents depending on the characteristics of TAZ. A more precise understanding can be achieved by comparing the suitability of the NB model and the GWNBR model for predicting accident counts. In the NB model, the 65 and older population (coefficient value: 6.723) is interpreted to increase bicycle accidents. However, in the GWNBR model, the coefficient values for the 65 and older population range from a minimum of − 0.326 to a maximum of 11.486. This result suggests that as the population aged 65 and older increases, there are TAZs where bicycle traffic accident counts decrease. Among 424 districts in Seoul, only one district, Banghwa 2-dong, displayed results contrary to the general trend. In such areas, it can be interpreted that there is no need to create a bicycle-friendly environment for the elderly. In this way, GWNBR, unlike the random parameter model, has the advantage of determining the sign of coefficients by TAZ. In Korea, local buses operate on local roads connected to residential areas. This characteristic forms the basis for a complementary means, where TAZs with high local bus usage exhibit reduced bicycle usage. If local buses and bicycles complement each other, it is generally expected that TAZs with high local bus usage will reduce bicycle accidents. However, it was found that in 129 out of 424 districts (30%) in Seoul, as local bus usage increased, bicycle accidents also increased. In this way, the GWNBR model, unlike traditional NB model or random parameter models, allows for the consideration of spatial heterogeneity and provides specific insights into what influences occur in different spaces. Therefore, GWNBR is the most suitable approach for spatially understanding the causes of bicycle accidents and enables various interpretations as follow.

**Figure 5:**
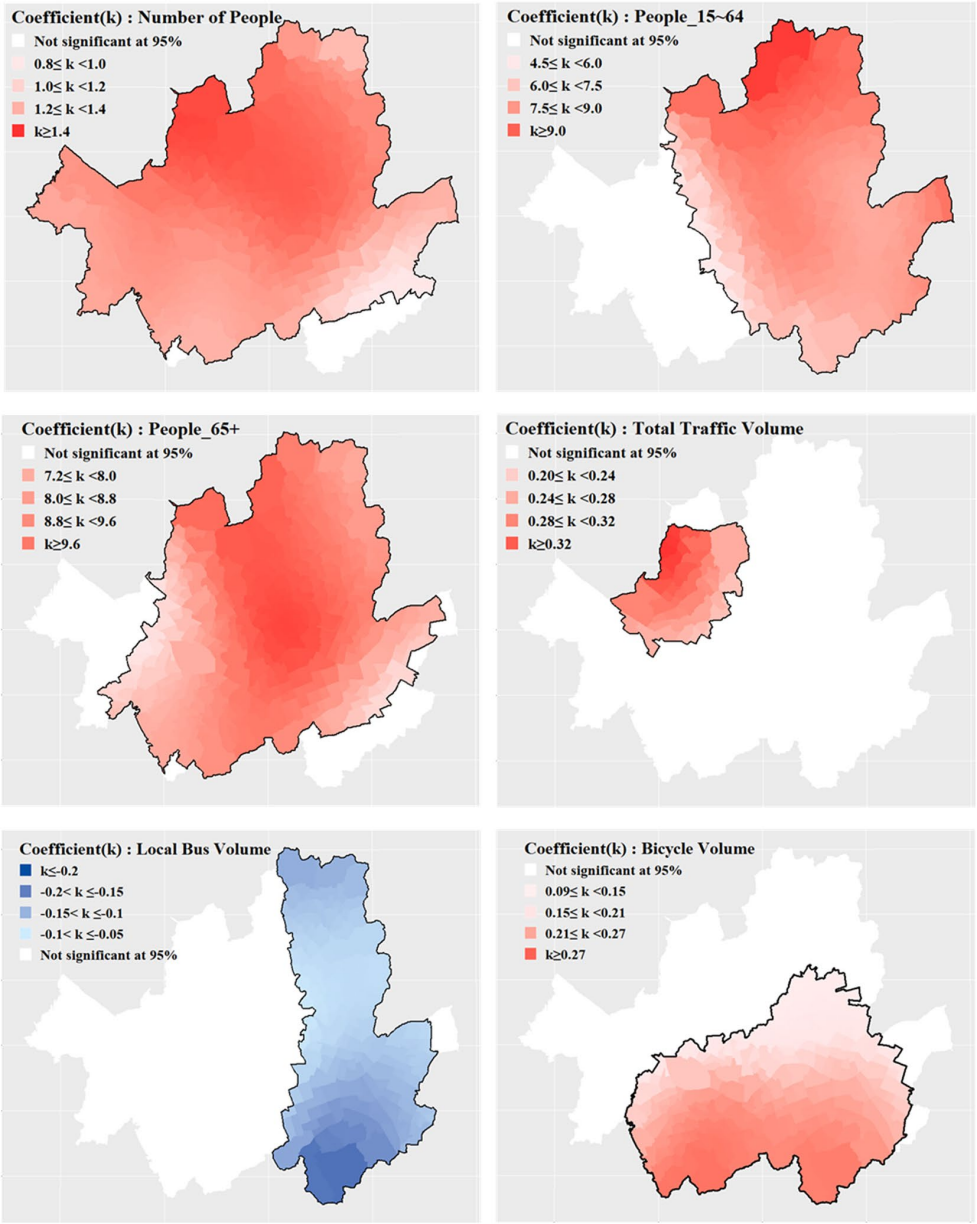
Significant at the 95% confidence level of TAZ by variables (bold boundary).

**Figure 6:**
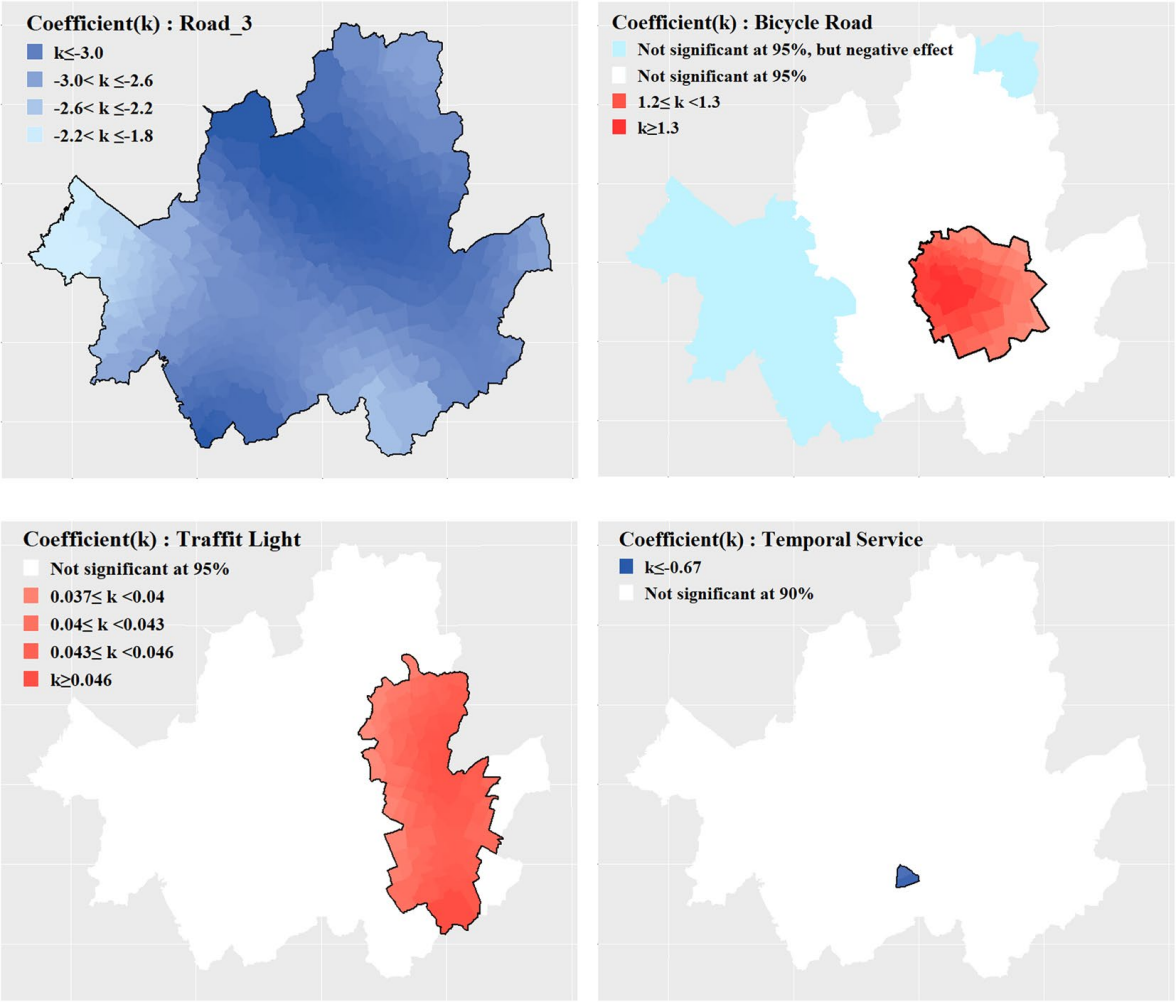
Significant at the 95% confidence level of TAZ by variables (bold boundary, except Temporal Service-90% confidence level of TAZ).

**Figure 7:**
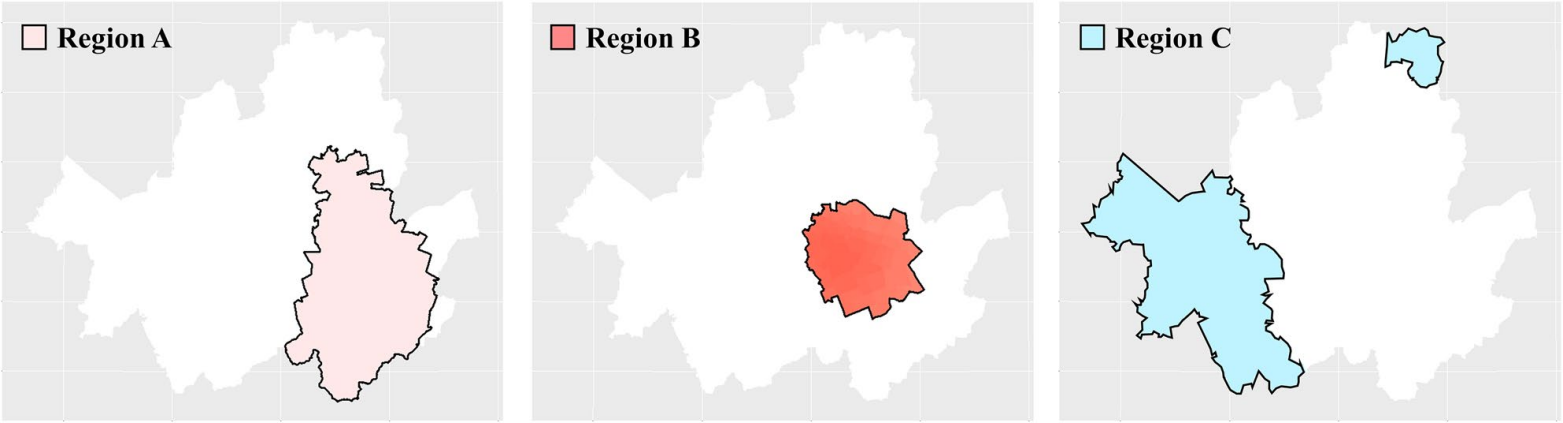
Locations of region A, B and C.

Furthermore, as this model can assess the statistical significance of coefficient by variables at a specified significance level, it can be performed interpretation for significant variables within a specific significance level. The results of the optimal model, including the coefficients for each geographical area, are visualized in Figs. 5, 6 and 7. This model takes into account spatial heterogeneity and facilitates the identification of variable significance on a regional basis. Thus, it enables to interpret and compare each variable by TAZs. Figures 5 and 6 are  visualization of the results regarding the impact of variables on bicycle traffic accidents within a specific significance level (95% confidence level), ensuring statistical validity. Figure 7 represents the regions that need further examination based on the derived results. TAZs outlined with thick black lines represent cases where the |t-value| of the corresponding variable exceeds 1.96, indicating that the variable has a statistically significant impact on TAZ at a 95% confidence level.

Regarding the analysis of population and population composition ratios, it was found that for the majority of TAZs (97.8%), an increase in population was associated with an increase in bicycle accidents. Within the analysis scope, approximately 69.6% of TAZs showed a positive correlation between the 15 and 64 age group population ratio and bicycle accidents, with a higher ratio leading to more accidents. Similarly, about 86.6% of the TAZs exhibited a positive correlation between the population ratio of those aged 65 and above and bicycle accidents. These findings align with previous study results^8^. However, as shown in Figs. 5 and 6, there are particular TAZ where variables do not have a significant impact on the frequency of bicycle traffic accidents.

In terms of traffic volume, only some TAZs (11.3%) concentrated in the northwest region exhibited a positive relationship where higher overall traffic volume was associated with more bicycle accidents. However, for over half (54.5%) of the TAZs located in the southern part of Seoul, an increase in bicycle traffic volume corresponded to an increase in bicycle accidents. This outcome can be interpreted based on the spatial heterogeneity of the relationship between traffic volume and bicycle accidents, as previously suggested by other studies^12,40^. Moreover, among TAZs accounting for 40.6% of the entire analysis scope and located in the eastern part of Seoul, an increase in local bus traffic volume was linked to a decrease in bicycle accidents. Notably, Region-A (23.3% of TAZs) showed that an increase in local bus traffic volume corresponded to a decrease in bicycle accidents, and an increase in bicycle traffic volume corresponded to an increase in bicycle accidents. This suggests a statistical inference of substitutability between local buses and bicycles in Region-A. To explain the potential substitution relationship between local buses and bicycles observed in Region A, which differs from other TAZs, an additional analysis that compare characteristic by each groups was conducted. The analysis results are presented in Table 8.

**Table 8:** Results of additional analysis between Region-A with others.

Type	Region A	Other TAZs
Average traffic volume of local buses (veh/day)	2255.44	3426.56
Average traffic volume of bicycles (veh/day)	1460.96	1043.98
Difference in average traffic volume between local buses and bicycles (veh/day)	794.48	2382.58
Average percentage of road in slope < 3% (%)	82.90	69.87
Average percentage of bicycle road (%)	13.05	8.41
Average public transportation spatial service index	0.86	0.85
Average public transportation temporal service index	0.95	0.97

Firstly, the difference in traffic volume between local buses and bicycles is lower in Region-A compared to other TAZs. Region-A attracts more bicycle users than local buses. Notably, roads in Region-A with slopes ranging from − 3 to 3% constituted an average of 82.9% of the total road length, whereas in other regions, this average was 69.9%. In the case of Region A, characterized by relatively flat road gradients, which contribute to an increase in bicycle traffic volume, the result verified that the previous research findings, which had a positive impact on bicycle users, were consistent^16^.

Additionally, the mean proportion of dedicated bicycle lanes in Region-A was 13.1%, whereas in other regions, it was 8.4%. The installation rate of bicycle lanes exhibited a pattern similar to bicycle traffic volume. Indeed, Region A had a higher average bicycle traffic volume compared to other TAZs, interpreted as a relatively higher installation rate of bicycle lanes to meet the demand of these users.

Finally, transit service data was compared and examined to investigate whether differences in public transportation service levels are the primary cause of variation. The results revealed that there is no significant difference in public transportation service levels among the regions. If Region-A had significantly lower transit service levels compared to other TAZs, it could be inferred that an increase in bicycle accidents may be attributed to increased bicycle traffic due to the absence of local buses and subway stations. However, the analysis indicated that the difference in service levels between these two regions is subtle. Therefore, it can be interpreted that the observed outcomes are not solely attributable to disparities in public transportation accessibility.

These findings indicate that in TAZs with lower average road inclines and a higher proportion of bicycle lanes, there is an increased frequency of bicycle usage, potentially leading to a higher likelihood of bicycle accidents. Furthermore, these findings also illustrate that bicycles are in a substitution relationship with local buses not public transportation, including subways and general buses in the specific TAZs.

In the context of road slope, the study revealed that across all areas, an increased proportion of roads with slope sizes between 3 and 6% was associated with a decrease in accidents. As road slope has a significantly negative impact on cyclists’ route choice^21^, the observed decrease in accidents might be attributed to the reduced exposure to road segments with slopes and a decrease in accidents associated with such segments.

Previous research has also found a positive relation between increased bicycle lane installation and more accidents^16^. The results of this study also revealed that bicycle accidents increase as the proportion of bicycle lane installations increases. However, appropriate bicycle infrastructure installations are known to ensure cyclists’ right of way and provide a safer environment, ultimately leading to a reduction in accidents^15,41^. Indeed, bicycle lanes in Seoul are installed considering several of factors such as traffic volume, request of citizens, road geometry^42^. Therefore, interpreting in fragment that a high proportion of bicycle lane installations as having a higher risk of bicycle accidents without considering other factors including bicycle exposure could lead to an incorrect conclusion. The optimal model proposed in this study is a local model that takes into account spatial heterogeneity by constructing different accident prediction models for each TAZ. In other words, as the impact of bicycle lane installation on bicycle traffic accidents varies across TAZs, conducting additional analysis based on the coefficients of variables representing the proportion of bicycle lane installations can help derive appropriate bicycle lane installation strategies.

Although the 110 TAZs (25.94%) where bicycle accidents appeared to decrease with an increase in bicycle lane installation were not statistically significant, TAZs where accidents increased with an increase in bicycle lane installation were defined as Region-B, and TAZs where accidents decreased with an increase in bicycle lane installation were defined as Region-C. Subsequently, the analysis was conducted. Table 9 shows the additional analysis results.

**Table 9:** Results of additional analysis between Region-B with Region-C.

Type	Region B	Region C
Average traffic volume of bicycles (veh/day)	1460.04	1328.35
Average bicycle road network (km)	3.20	3.14
Average percentage of installation protected bicycle path (%)	7.86	41.01
Average percentage of installation bicycle lane on road (%)	64.24	53.52
Average percentage of installation shared bicycle and pedestrian path (%)	7.24	1.19
Average percentage of installation shared bicycle road (%)	20.66	4.28
Average road width (m)	6.9	6.4
[Protected bicycle path] Bicycle lane separated from the road and sidewalk by barriers or dividers, allowing only bicycles [Bicycle lane on road] Bicycle lane on the roadway designated exclusively for bicycles [Shared bicycle and pedestrian path] Bicycle lane on the sidewalk where both pedestrians and bicycles share the space [Shared bicycle road] Bicycle lane on the roadway where both vehicles and bicycles can travel without a physical separation		

Relatively high bicycle traffic volume results in higher number of bicycle accidents. However, the average bicycle traffic of the 424 existing TAZs in Seoul was 1139.38 (veh/day), confirming that both regions had relatively high bicycle traffic. Moreover, the differences in the extension and type of bicycle path were analyzed. It can be inferred that the extension of bicycle paths was analyzed at a similar level in both regions, and the extension of simple bicycle paths does not have an absolute effect on bicycle accidents only for some TAZs.

Unlike the extension of bicycle paths, when it delved into the type of bicycle paths, there was a significant difference in the extension according to the type of bicycle path. It can be seen that the installation rate of protected cycle paths that provide a separate space from the right of way of cyclists in Region C is much higher than that of Region B, and the installation rate of shared cycle paths that are installed and operated on roadways is lower than that of Region B.

These relationships are guaranteed reliability by the characteristics of Region B, which has a relatively wide average width of the road. This is because that region is a main Central Business District in Seoul and has various transportation modes and large traffic volumes. In order to minimize inconvenience to the significant number of pedestrians using sidewalks and utilize the relatively wide road width or number of lanes, there is a tendency to operate shared cycle roads in the area. This study suggests the need for further analysis considering different types of bicycle paths based on bicycle usage types, which would allow for identifying road types that tend to increase bicycle accidents and implementing additional analysis for such road types in the future.

Moreover, this study found that within the 95% confidence level, an increase in the number of traffic signals per unit length (1 km) is associated with an increase in accidents for 20.94% of the total analyzed areas. These areas are likely to experience higher chances of bicycle-related accidents at intersections where signals are installed and on certain single roads. Consequently, in such areas, safety measures should involve educating cyclists about proper crossing behavior and enhancing enforcement of traffic signal violations.

Finally, only 0.54% of the analyzed areas demonstrated a tendency for bicycle accidents to decrease within a 90% confidence level as the Transit Temporal Service level increased. These areas are closely adjacent to region A, where bicycle and local bus services can be considered substitutes. This result implies that for modes of public transportation such as local buses, the frequency of operation and intervals between services (temporal service) may have a greater impact on bicycle accidents compared to the mere distance to public transportation infrastructure (spatial service).

Based on these findings, policymakers for each TAZ can propose policies and plans to establish a safer cycling environment. For instance, in the case of Region B, where bicycles and local buses are substitutes and an increase in bicycle lane installations is associated with more accidents, the following safety measures could be suggested.

1. Increase the frequency of local bus operations to promote public transportation use and reduce bicycle accidents.
2. Expand the installation of protected bicycle paths on roads with gentle slopes to ensure cyclist safety.
3. Implement educational programs targeting all age groups to raise awareness about crossing behavior and traffic signal compliance.

This study also proposes tailored bicycle safety measures considering the inherent characteristics of each region.

## Conclusion

This study has several significant findings in comparison to previous research on factors influencing bicycle traffic accidents. First, it improved upon the limitations of traditional regression models (fixed parameter) that cannot account for spatial heterogeneity. It used a spatial analysis model, specifically GWNBR, to demonstrate that the factors influencing bicycle traffic accidents vary by each TAZs when using spatial analysis models. Though it is generally considered that bicycle traffic accidents increase with higher traffic volume, bicycle traffic volume, and the presence of bicycle lanes, the results of this study show that these considerations do not hold true uniformly across all TAZs. For instance, an increase in bicycle lane length tended to correlate with an increase in accidents; however, in areas with a higher proportion of dedicated bicycle lanes, there was a tendency for accidents to decrease as bicycle lane length increased. This suggests a need to apply bicycle accident impact factors based on specific types of bicycle path infrastructure. Though it was not possible to determine the specific characteristics of individual TAZs that influenced the significance of variables, the presence of spatial heterogeneity was evident. These results indicate that using spatial models instead of traditional regression models allows policymakers to develop more flexible and proper measures for reducing bicycle accidents.

Second, the study collected and applied variables that were not considered in previous research on bicycle accident impact factors. Instead of using terrain slope, which was used in previous research, this study calculated and applied road-link-specific slopes. Additionally, it collected and applied data on local bus traffic volume, considering that bicycles can serve as an alternative mode of transport for local buses. Furthermore, this study collected data on the level of public transportation services, including temporal and spatial accessibility, assuming that bicycles would be used more frequently in areas with limited public transportation services. The analysis of these variables, distinct from previous research, yielded significant results. It was found that as road slope increased, bicycle accidents decreased across all areas of Seoul. In some areas, bicycles and local buses acted as alternative modes of transport, and in certain regions, a decrease in public transportation service levels was associated with an increase in bicycle accidents.

These results suggest that policymakers, based on the findings of this study, can consider different factors with significant impacts on bicycle accidents in each TAZ, rather than implementing uniform bicycle accident safety measures. Taking into account the spatial characteristics and interactions between accident occurrence and influencing factors is expected to be much more effective in preventing bicycle accidents.

However, it has two main limitations. First, the utilized traffic volume data does not consider traffic movements within each TAZ. The traffic volume data used reflects traffic flows between TAZs, which means that for some modes of transportation used for First-Mile and Last-Mile connections, there might be discrepancies between the actual values and the data used. Second, although the study incorporated spatial heterogeneity into the model, there were limitations in exploring the various causes leading to heterogeneity. Though the interpretation of results from the optimal model indirectly suggests the occurrence of heterogeneity due to the types of bicycle lanes installed, it has limitations in explicitly identifying the diverse causes of spatial heterogeneity. As traffic accidents result from various potential factors, identifying the causes of heterogeneity could lead to the development of appropriate measures for possible accidents in the future.

## Data Availability

The data used in this study can be obtained from the websites mentioned in the paper. However, for accident data that may contain some personal information, could be challenging.
